# Light People: Professor Guangcan Guo

**DOI:** 10.1038/s41377-022-01045-7

**Published:** 2023-01-01

**Authors:** Hui Wang

**Affiliations:** Changchun Institute of Optics, Fine Mechanics and Physics, Chinese Academy of Sciences, 3888 Dong Nan Hu Road, Changchun, 130033 China

**Keywords:** Photonic devices, Quantum optics

## Abstract

The Nobel Prize in Physics 2022 was awarded to French physicist Alain Aspect, American physicist John F. Clauser, and Austrian physicist Anton Zeilinger, in recognition of their contributions to the research of quantum information, a clear indication that we are entering the era of quantum information.

In China, a scientist has played herald in quantum research since as early as the 1980s. He introduced quantum optics and quantum informatics to China, a virgin land in terms of quantum research back then. That scientist is Academician Guangcan Guo.

Out of personal interests and his good sense as a scientist, Prof. Guo took it upon himself to take on quantum optics when little was known about it in China. He trained many students and launched China’s first academic conference on quantum optics, which helped to accelerate the development of quantum optics in China.

In the early 1990s, he took on another challenge, shifting his research focus to the emerging subject of quantum informatics. His work helped to establish China’s current leading position in this interdisciplinary field. Today, China leads the world in quantum USB drives and quantum chips.

Now in his 1980s, Prof. Guo is still working tirelessly at the front line of scientific research, doing science popularization, and teaching students. He is ready for the third big challenge of his life—to reveal the mysteries of the classical world and the quantum world.

Next, please follow “Light People” into the mysterious “quantum world”, and feel the beauty of science.

## Abstract (web summary)

Prof. Guangcan Guo took it upon himself to delve into quantum optics and quantum informatics, which helped to accelerate the development of quantum science in China.

## Graphical abstract

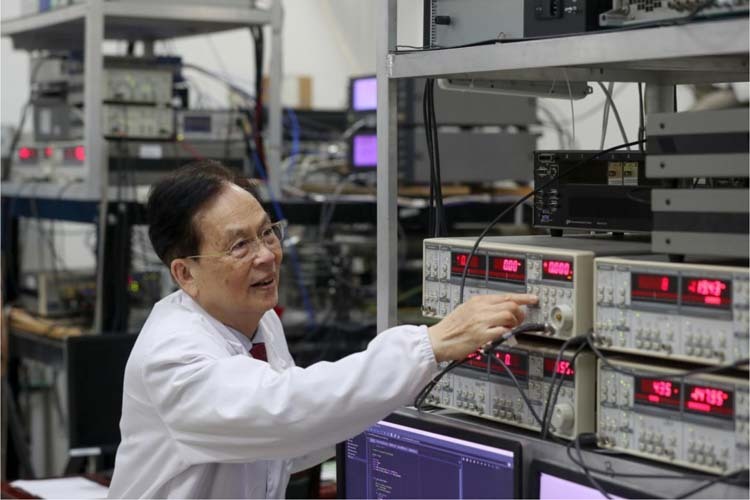

**Biography:** A trailblazer and founder of quantum optics as well as quantum information science in China, Guangcan Guo is an academician of the Chinese Academy of Sciences (CAS), academician of the Third World Academy of Sciences (TWAS), professor of the University of Science and Technology of China (USTC), and former president of the Chinese Optical Society (COS). In 1965, Prof. Guo graduated from USTC and was offered a teaching position there. From 1981 to 1983, he studied at the University of Toronto as a visiting scholar. He was elected an academician of CAS in 2003 and a member of TWAS in 2009. In the 1980s, Prof. Guo took the lead in introducing the theoretical system of quantum optics to China. In the 1990s, he took the lead in focusing on the field of quantum information and undertook the national “973 Plan” project, which led to the positive development of quantum information science in China. He put forward the principle of probabilistic quantum cloning, deduced the maximum cloning efficiency, and successfully experimentally developed the probabilistic quantum cloning machine and the universal quantum cloning machine. In 2021, Prof. Guo won the second prize in the 2020 National Natural Science Award for his Research on Fundamental Problems of Quantum Physics Based on Quantum Information Technology.

**Figure:**
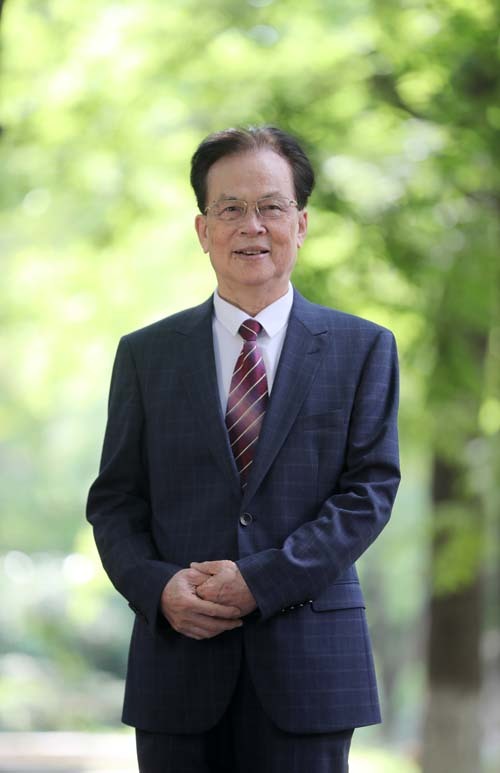

**1. You are a pioneer and leading researcher in the field of quantum research in China, so could you tell us briefly what your research focuses are?**

Prof. Guo: My research journey into quantum science began in the early 1980s. In the first ten years, I mainly focused on the basics of quantum optics. In the early 1990s, I stepped into the field of quantum information. I gradually built up a vigorous research team and established China’s first provincial-level key laboratory on quantum information. My main research directions and areas include the following:

1. Quantum computing. Developing special quantum simulation and general quantum computers based on semiconductors, superconductors, ion traps, and optical systems;
2. Quantum entanglement networks. Develop quantum light source, quantum channel, quantum memory, etc. Quantum relay and quantum USB drive are realized to construct large-scale quantum networks;
3. Quantum precision measurement. High-precision quantum sensing and quantum detection devices are developed by using solid-state spin and cold atom ensembles. Development of integrated quantum chips;
4. Practical quantum cryptography. Develop efficient, practical, and secure quantum cryptosystems and explore new quantum cryptosystems;
5. Fundamental questions of quantum physics. The basic problems of quantum information and quantum physics are studied theoretically, and quantum information technology is used for experimental exploration.

**2. You and your colleague Luming Duan published some of the earliest papers on quantum research by Chinese physicists. In a paper published in** ***Physical Review Letters*** **in 1997, you proposed a scheme to “preserve coherence in quantum computation by pairing quantum bits”. Then one year later, you proposed the probabilistic quantum cloning principle, known as the “Duan-Guo cloning machine”, which caught international attention. Yet I’m sure the road to academic fame is not all smooth sailing, so what obstacles have you encountered over the years, and what significance did overcoming those obstacles have on the development of quantum optics?**

Prof. Guo: Both the opening of quantum optics and the subsequent research on quantum information was “unpopular” in domestic academic circles at that time, as they were difficult to understand or support, and even labeled as “pseudoscience”. After 20 years of being “lonely and helpless”, the first “973 Plan” project of the Ministry of Science and Technology in 2001 brought real changes. It would be impossible to obtain important progress in Quantum information technology without the support of national funding.

My motivation for studying quantum optics is primarily a personal interest in quantum science, but when I saw what significance quantum information technology has to the building of national strength and our national rejuvenation, especially how quantum computing will become the focus of international strategic competition, I felt I had a responsibility to gain understanding and support from academics and policymakers despite a lack of understanding from the public. China needed to be propelled into this new interdisciplinary competition as soon as possible, so the voice of the Chinese people could be heard. Since then, the Ministry of Science and Technology has been paying attention to and supporting the development of quantum information. As the chief scientist of the first “973 Plan” project, I tried my best to win over relevant academic teams in China. This project has trained five academicians in the field of quantum information. Therefore, I believe that 2001 was a turning point in the development of quantum information in China.

**3. In 2000, your proposed scheme on how to generate a two-atom entangle state with cavity QED was again highly acclaimed. Serge Haroche, the French physicist, was awarded the 2012 Nobel Prize for Physics for proving that it is possible to isolate single ions and photons. What kind of significance do you think this method has?**

Prof. Guo: French scientist Serge Haroche won the 2012 Nobel Prize in physics for being the first to experimentally manipulate a single atom, thus ushering in a new era of “quantum regulation”. Our theoretical scheme is an efficient way to fabricate the entanglement of two atoms. Haroche used his experimental methods and techniques to fabricate a two-atom entangled state using our scheme. This is one example of the success of his quantum manipulation.

**4. One of your recent papers is on the successful loophole-free experimental verification of the Leggett-Garg inequality in a millimeter-level macro world, which is believed to be of great significance to the second revolution of quantum mechanics. From quantum optics to quantum information and quantum mechanics, how are you able to transit through these fields with such graceful ease? And how will this experiment result impact the development of quantum mechanics?**

Prof. Guo: Quantum optics is a full quantum theory that studies the interactions between light and matter, and it applies quantum mechanics to the optical field. In the 1980s, this subject was already well-researched, but domestically in China, it was still relatively unknown due to various reasons. What we did was just to fill in the gaps in China, applying quantum optics to all areas of physics studies, while developing a quantum optics research and teaching team in the meantime. Quantum information is the application of quantum mechanics to the field of information. In the early stages of the development of this new interdisciplinary subject, most of the information carriers were light fields. We applied well-established quantum optics theory to the research of quantum information in time, and soon made a series of important progress. The development of quantum information gradually extended to the field of atoms and condensed matter. When the development of quantum information technology was more mature, we applied this technology in turn to the study of the mysteries of the quantum world and discovered many new quantum phenomena. One of the results is to verify LG inequality without loopholes. These researches have improved our understanding of quantum mechanics and promoted further research.

**5. Quantum USB drive is a very popular concept these days. How does it work? What kind of changes will it bring?**

Prof. Guo: The working principle of the “quantum USB drive”: store quantum information which is to be transmitted in a “USB drive”, and then the “quantum USB drive” is transported to the target receiver by traditional transportation means, such as high-speed rail, car, plane, etc., where the information in the “quantum USB drive” is extracted. This remote quantum information transmission method can make up for the lack of optical fiber remote quantum communication. It does not need an entanglement source or quantum relay and can be transmitted to places without optical fiber ports. It is a novel remote quantum communication scheme.

Of course, to achieve such remote quantum communication, the “quantum USB drive” must meet several performance indicators: the storage time should be more than one hour. At present, our coherent optical storage has reached 1 hour and is expected to reach 25 hours, further realizing single-photon storage. There should be sufficient memory storage modes; The fidelity should be high enough. Further development would involve entangling two quantum USB drives, sending one to the other. This would ensure the security of the transmission since even if the drive was stolen by someone eavesdropping, they would not be able to retrieve any of the transmitted information.

**6. The quantum optics conference which you proposed and organized, the first of its kind in China, is attended by more than 500 people now each time, much more impressive than the dozen or so people when it was first launched. It offers a wonderful platform for quantum researchers to network, learn and exchange ideas. What gave you the idea for such a conference? Has its development been to your expectation?**

Prof. Guo: The reason why I initiated and organized the “Quantum Optics Conference” in the 1980s was that I realized that “quantum optics” was an important fundamental discipline, extremely important not only to the field of optics but also to the entire world of physics. It must be introduced to Chinese academics as soon as possible to promote the development of quantum optics in China. In 1984, the seed of “quantum optics” was planted in Langya Mountain, Anhui Province. Since then, a national academic conference has been held every two years, attracting more and more young people to join the quantum optics team. Especially after the birth of quantum information, quantum optics has played a pivotal role in it. The research team has expanded rapidly, one after another universities started to offer courses in “quantum optics”. “Quantum optics” quickly took root in China and demonstrated a vibrant prospect, which reached my original expectation.

**7. Quantum research is widely recognized now as one of the most significant fields of physics studies. To what do you think it owes its fast development?**

Prof. Guo: “Quantum information” developed so rapidly because it provides human society with a new information technology whose performance far exceeds current information technologies. Once quantum technology is widely used, human social productivity can be raised to new levels. It is the great need for human development that promotes the rapid development of quantum information.

**8. When you took the decision to study quantum optics, many apparently tried to talk you out of it. Quantum was certainly not a household word at the time. What made you choose it as your life-long work? Looking back now, what do you think are your biggest gains?**

Prof. Guo: When a discipline is just emerging, most people do not understand it, so they may question or even oppose it; this is a normal phenomenon. In retrospect, I was deeply attracted by “quantum information” as soon as I came into contact with it. I soon realized that this new subject had great development prospects, and I threw myself into it without hesitation. The more I studied it, the more I realized its great potential. I gradually grasped the pulse of the development of quantum information technology and was able to make arrangements in advance, which led to timely cutting-edge achievements and the complete layout of the current laboratory.

**9. Over the years, you’ve had more than 80 Ph.D. students. What capabilities do you most want your students to develop, and what are your expectations for them?**

Prof. Guo: Scientific research should be accumulated and inherited. I believe that the development of “quantum information” needs the joint efforts of several generations, so I pay great attention to the training of students. More than 80 doctoral students have been trained, among which 5 have received the honor of having their dissertations ranked among the top 100 of their year in China. I pay special attention to (1) Arousing students’ enthusiasm for scientific research, and taking scientific research as a lifelong career; (2) Selecting international cutting-edge subjects for students and encouraging students to make world-class achievements; (3) Trying to create good scientific research and living conditions for students; (4) Ensuring that the laboratory has a good academic atmosphere and train students in the scientific spirit of seeking truth from facts. Taking “savour science” as our motto, I hope to cultivate world-class talents on Chinese soil.

**Figure:**
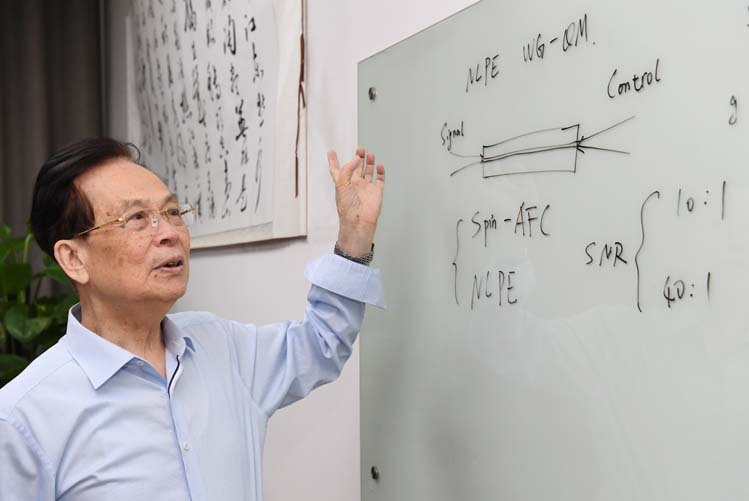
Academician Guangcan Guo giving a lecture

**10. In 2017, you jointly launched Origin Quantum Computing Technology Co. Ltd. with your former student Prof. Guoping Guo of the University of Science and Technology of China (USTC). Your company is the first dedicated quantum computing developer in China and has made many breakthroughs. What can you tell us about your company? Also, how do you think research and industry should be linked?**

Prof. Guo: Origin Quantum Computing Technology Co. Ltd was founded to turn the results of more than a decade of quantum computing research in our laboratory into practical, engineered quantum computers. The company’s leading engineer is a Ph.D. in quantum computing trained by us. During the past five years, the company has independently developed 24- and 64-bit superconducting quantum computers (Origin Wuyuan), which have been delivered to users. It has also developed the first Sinan operating system in China, as well as quantum devices such as the “Origin Heavenly Machine” and quantum computing measurement and control systems. The quantum computing cloud platform has been officially launched, with thousands of registered users up to now. This is the first full-stack engineering quantum computing company in China and has a certain influence in the world.

The value of quantum information technology lies in providing excellent quantum technology and devices for human society. When we find some new principles and methods in the laboratory, we should move toward the practical direction as soon as possible. But the process must address a number of technical and engineering issues. This requires a large number of quantum engineers. Schools and research institutes can only be staffed by quantum scientists, while establishing enterprises can train a large number of quantum engineers. The organic combination of the two forces is the best way to promote the research and development, and application of new devices. This is our experience.

**11. Though celebrating your 80**^**th**^ **birthday this year, you are still hard at work. What’s the secret to your health and vigor?**

Prof. Guo: Though over 80, I am still active on the front line of scientific research, mainly because of my inner dedication to scientific exploration. The wonderful quantum world will continue to amaze and entice you with endless new phenomena, thus further stimulating your curiosity. This kind of exploration is not limited by age; as long as the spirit is in, the pace of exploration will not stop.

**Figure:**
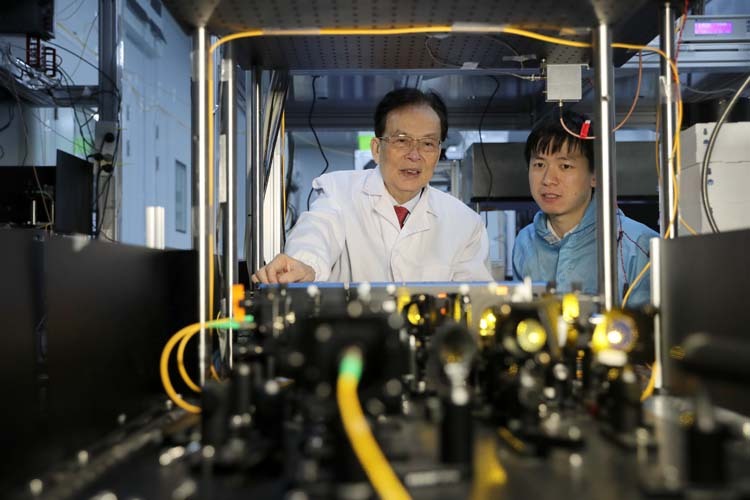
Academician Guangcan Guo and Dr. Chao Liu discussing the quantum relay experiment in the Solid State Quantum Memory Laboratory

**Figure:**
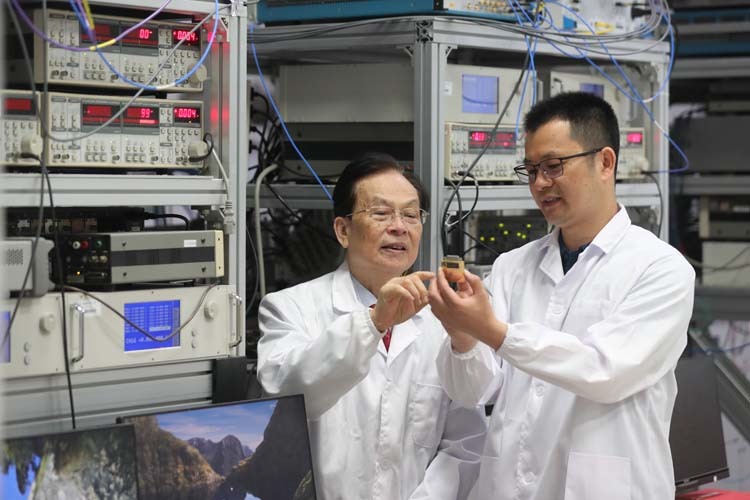
Academician Guangcan Guo talking with Prof. Gang Cao at the Semiconductor Quantum Chip Laboratory

**Figure:**
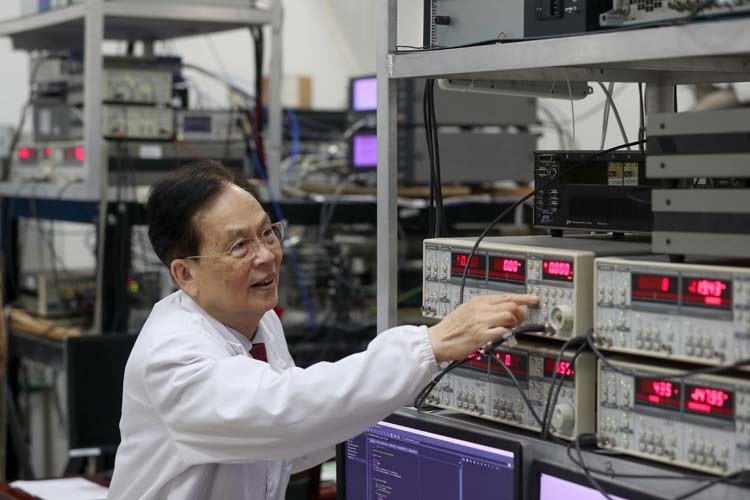
At the age of 80, Academician Guangcan Guo is still active on the frontline of scientific research

**12. What, in your opinion, are characteristics a good science worker must have?**

Prof. Guo: The necessary qualities for a scientific researcher should be: passion for exploring nature; good scientific spirit and discipline; indomitable will, and never giving up.

**13. What’s your motto in life?**

Prof. Guo: Treat people with sincerity, and face work with earnestness.

**14. Do you have any hobbies? What are they?**

Prof. Guo: Interests and hobbies: reading, listening to music. When young, volleyball, badminton, bowling, and long-distance running. When old, Baduanjin, walking.

**Figure:**
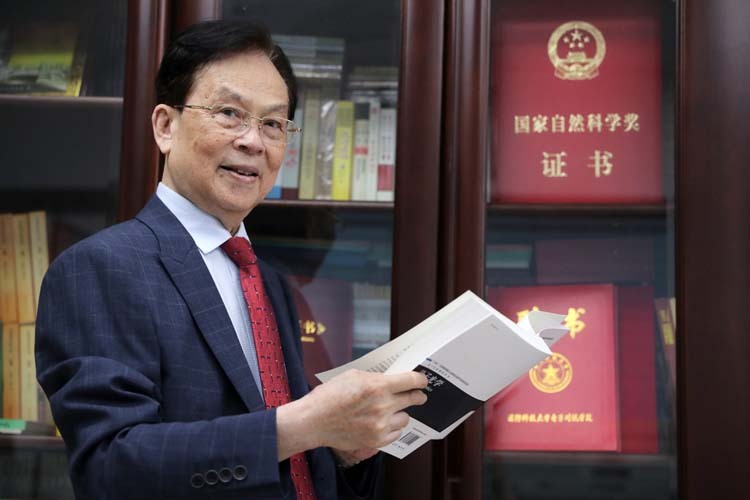
Reading is Academician Guangcan Guo’s greatest interest

**Figure:**
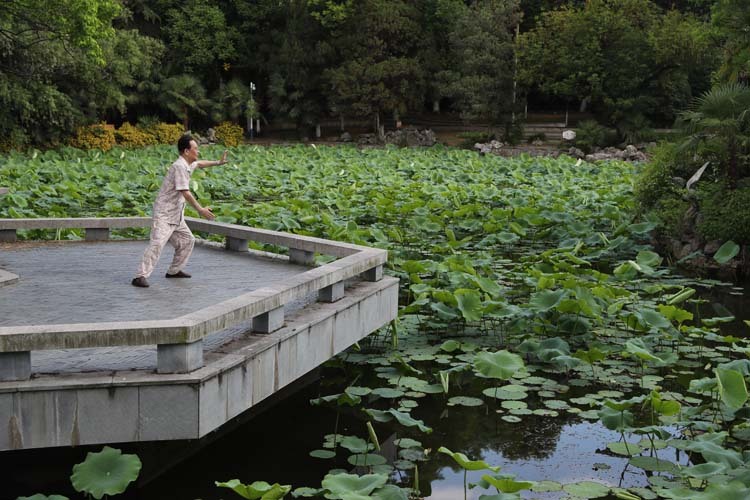
Academician Guangcan Guo practicing Baduanjin (ancient Chinese Qigong skill)

**15. What advice and expectations do you have for today’s young researchers?**

Prof. Guo: Today’s young researchers should cherish the hard-won research environment, have the enthusiasm to pursue scientific research, and combine their personal goals with the needs of the country. We should have the scientific spirit of seeking truth from facts and seek to contribute our lives to mankind and the country.

